# Silencing GDI2 inhibits proliferation, migration and invasion of colorectal cancer through activation of p53 signaling pathway

**DOI:** 10.1016/j.heliyon.2024.e37770

**Published:** 2024-09-13

**Authors:** Wen-Ting Ou, Rong-Jian Tan, Jia-Wei Zhai, Li-Jun Sun, Fei-Peng Xu, Xian-Jin Huang, Zhen-Hao Quan, Cai-Jin Zhou

**Affiliations:** Affiliated Hospital of Guangdong Medical University, No. 57, South of Renmin Avenue, Zhanjiang, 524001, China

**Keywords:** Colorectal cancer, GDI2, Transcriptomics, p53 signaling pathway

## Abstract

**Objective:**

To investigate the effect of silencing GDP dissociation inhibitor 2 (GDI2) on colorectal cancer development and possible mechanisms based on transcriptomic analysis.

**Methods:**

The differences in the expression levels of GDI2 in normal colorectal tissues and tumor tissues of colorectal cancer (CRC) patients were detected. The correlation of GDI2 expression levels with survival and clinical characteristics of CRC patients was analyzed. The effects of GDI2 expression levels on the biological functions of CRC cells were examined by CCK-8 assay, plate clone formation assay, wound healing assay, and Transwell assay. The effect of GDI2 on the proliferation and growth of xenograft tumors was investigated by a xenograft tumor model of CRC in nude mice. Based on transcriptomics, we explored the possible mechanisms and associated pathways of the effect of silencing GDI2 on CRC cells. Cellular experiments and Western blot assays were performed to verify the potential mechanisms and related pathway of GDI2 action on CRC.

**Results:**

The expression levels of GDI2 in CRC tissues and cells were higher than those in normal tissues and cells. The expression level of GDI2 correlated with clinical characteristics such as lymphatic metastasis, tumor stage, tumor volume, and lymphocyte count. Silencing of GDI2 reduced the proliferative activity and migration and invasion ability of CRC cells, as well as inhibited the proliferation of CRC xenograft tumors. The differentially expressed genes were significantly enriched in biological processes such as cell cycle arrest and the p53 signaling pathway after GDI2 silencing. The percentage of G0/G1 phase cells in CRC cells was increased after silencing GDI2 as verified by flow cytometry. RAB5A was highly associated with the p53 pathway and could interact with TP53 via the ZFYVE20 protein. The mutual binding between GDI2 protein and RAB5A protein was verified by immunoprecipitation assay. Silencing GDI2 while overexpressing RAB5A reversed the reduced proliferation, migration, and invasion ability as well as cell cycle arrest of CRC cells. Meanwhile, the addition of p53 signaling pathway inhibitor Pifithrin-α (PFT-α) also reversed the biological effects of silencing GDI2 on CRC cells. The p-p21 and p-p53 protein expression levels were significantly greater in the sh-GDI2 group than in the sh-NC group. However, the p-p21 and p-p53 protein expression levels were reduced after silencing GDI2 while overexpressing RAB5A.

**Conclusion:**

Silencing GDI2 activates the p53 signaling pathway by regulating RAB5A expression levels, which in turn induces cell cycle arrest and ultimately affects the proliferative activity, migration, and invasive ability of CRC cells.

## 1 Introduction

Colorectal cancer (CRC) is a malignant tumor of the gastrointestinal tract originating from the colon or rectum and is the second leading cause of cancer deaths worldwide [1]. CRC develops slowly, has a complex and diverse pathogenesis, and can be caused by a variety of risk factors, including personal dietary habits, environmental factors, and genetic family history [2]. Common clinical symptoms include blood in the stool, iron deficiency anemia, abdominal pain, weight loss, and loss of appetite. Currently, the clinical treatments for CRC include surgical resection, radiotherapy, chemotherapy, and immunotherapy. The predominant tool is surgical resection, but the therapeutic efficacy is unsatisfactory, especially for patients diagnosed with stage 4 CRC, with a 5-year survival rate of less than 10 % [3]. In recent years, approximately 20 % of new CRC cases have been observed to involve metastatic disease [4]. For patients with metastatic disease, radiotherapy is generally the preferred treatment, but there are limited therapeutic drugs available for targeting tumors in clinical practice [5]. Although radiotherapy can effectively reduce the risk of CRC recurrence and increase overall survival rates, it can also have significant adverse effects, such as oral ulcers and gastrointestinal reactions [6]. The prognosis of CRC is related to the stage at diagnosis, with less than a 10 % survival rate when distant metastases occur. Therefore, it is crucial to develop reliable biomarkers with the ability to predict CRC metastasis.

GDP dissociation inhibitor (GDI) is a small GTP-binding protein in the Ras superfamily that regulates GDP-GTP exchange among RAb family members and affects the vesicular transport of substances between organelles [7]. The identified forms of GDI include GDI1 and GDI2. Specifically, the GDI2 gene, located at 10p15.1, consists of 76910 nucleotides and encodes a protein called GDI2 with a molecular weight of 51 KD(8). The GDI2 gene is upregulated in various cancers and can regulate biological functions, including tumor cell proliferation, apoptosis, migration, and cell metabolism [[9], [10], [11]]. Our previous study showed that dimethylhydrazine (DMH)-induced CRC rats exhibited enhanced mRNA expression levels of GDI2 [12]. However, the specific role of GDI2 in CRC development and its mechanisms remain unclear and warrant further investigation.

To investigate the role of GDI2 in CRC development, we conducted a series of in vitro cellular experiments and in vivo experiments based on previous studies. We aimed to elucidate its mechanism of action and related signaling pathways through transcriptome analysis, expecting to provide potential tumor markers for the prevention and treatment of CRC.

## 2 Materials and methods

### 2.1 Cell culture and transfection

NCM460, HCT116, and SW1116 cell lines were purchased from the Cell Bank of the Chinese Academy of Medical Sciences (Shanghai, China). Cells were cultured at 37 °C in DMEM medium (Four Seasons Biological Company, Hangzhou, China). Logarithmic growth stage HCT116 cells and SW1116 cells were used and transfected with short hairpin RNA (shRNA)or shRNA-GDI2. The three shRNA sequences targeting GDI2 (sh-GDI2#1, sh-GDI2#2, and sh-GDI2#3) and the negative control shRNA (sh-NC) were produced by GenePharma (Shanghai, China). Cells were transfected with Lipofectamine® 2000 transfection reagent (Invitrogen, USA). The sequence with the best inhibition rate was screened using quantitative real-time RT-PCR (qRT-PCR) and Western blot. Overexpression of RAB5A was performed by qRT-PCR of the complete coding fragment of RAB5A, and the fragment was ligated into the pcDNA3.1 vector to construct pcDNA3.1-RAB5A. Transfection of empty pcDNA3.1 was used as a negative control. For sh-GDI2+PFT-α group and PFT-α group cells, PFT-α (SY1065, Biolab, China) was dissolved in dimethyl sulfoxide (DMSO) before pro-use and needed to be added to the culture plate about 4 h before transfection.

### 2.2 CRC tissue specimens

Patients diagnosed with CRC at the Affiliated Hospital of Guangdong Medical University between January 2022 and February 2022 were collected through the case system of the inpatient department of the Affiliated Hospital of Guangdong Medical University. CRC tumor tissues from CRC patients were taken as the experimental group, and normal tissues adjacent to the cancer from the same patients were used as the control group for subsequent experimental analysis. All patients included in the study were diagnosed by cytological or histopathological methods, and all patients had not received chemotherapy or radiotherapy. All participating patients were informed and signed an informed consent form before the study. The study protocol was approved by the Ethics Committee of the Medical Center of the Affiliated Hospital of Guangdong Medical University (No. YJYS2022237).

### 2.3 Bioinformatics data analysis

Download RNAseq data and clinical information in grade 3 HTSeq-FPKM format from the TCGA website for the Colon Cancer (COAD) and Rectal Cancer (READ) programs. Meanwhile, normal control cases from the GTEx database were downloaded from UCSC XENA and processed uniformly by the Toil program. The level 3 RNAseq data from TCGA and GTEx in the format of HTSeq-FPKM were converted to TPM for subsequent analysis. Referring to the study by Liu [13], we tested whether the GDI2 gene was correlated with the clinical data. Clinical data and TCGA data were first processed, including age, gender, lymphatic invasion, Tumor Node Metastasis (TNM) stage, tumor stage, presence of metastasis, lymphocyte count, and tumor volume size. Differences in GDI2 gene expression between the control group and the cancer group were compared. Clinical symptoms were combined with gene and gene expression after all treatments. The gene risk values and clinical traits were correlated by the beeswarm package in R language. The correlation between genes and different clinical traits was determined based on the magnitude of the P-value. The clinical prognosis of GDI2 regarding overall survival (OS), progression-free survival (PFS), disease-free survival (DFS), and disease-specific survival (DSS) in CRC patients was assessed using the Survminer package concerning the experimental approach of Liu [14]. Survival curves were constructed using the Kaplan-Meier method. P values and hazard ratios (HR) with 95 % confidence intervals (CI) were derived by log-rank test and univariate Cox regression [15]. *P* < 0.05 was considered statistically significant.

### 2.4 qRT-PCR

The primers were designed according to the sequences of related genes included in GenBank and synthesized and purified by Shanghai Sankyo Biotechnology Co. The sequences of GDI2 primers are as follows, forward: 5′-ATTCCACAGAACCAAGTCAATCGA-3′, reverse: 5′-CCTCTCAGCTCCTTGGTTTCC-3'. The GAPDH primer sequences were as follows, forward: 5′-GGGCTGCTTTTAACTCTGGT-3′, reverse: 5′-TGGCAGGTTTTTTTCTAGACGG-3'. Total RNA was extracted from HCT116 and SW1116 cells. cDNA, SYBR Green PCR Master Mix (Applied Biosystems, Foster City, CA), and gene-specific primers were used for qRT-PCR. Reaction conditions: 95 °C for 30 s, 95 °C for 10 s, 72 °C for 15 s, 40 cycles. Using the 2^−ΔΔCT^ method, the relative expression of each target gene was calculated.

### 2.5 CCK-8 assay

Cell proliferation was measured using the Cell Counting Kit-8 (CCK-8) assay. CRC cells were seeded into 96-well plates for 24 h. Then cells were incubated in 10 % CCK-8 solution for 1 h, and the absorbance value was measured at 450 nm using a microplate reader. Each experiment was performed in triplicate.

### 2.6 Plate clone formation assay

HCT116 and SW1116 cells at the logarithmic growth stage were transfected for 48 h and then inoculated into pore plates after re-suspension. When visible clones are observed, the culture is terminated. Fixed with pure ethanol, and stained with 0.1 % crystal violet, images were collected, and the clones were counted directly with the naked eye.

### 2.7 Wound healing assay

HCT116 and SW1116 cells were collected and seeded into six-well plates. After the cell density reached the whole plate, the scratch wounds were made by dragging a 200-μl pipette tip across the monolayer. Samples were taken at 0 h,4 h,16 h, and 24 h time points, and the state of wound healing in each well was followed under a 50 × microscope at each horizontal straight-line marker.

### 2.8 Transwell assay

HCT116 and SW1116 cells at the logarithmic growth stage were taken, set up in groups, and transfected or administered. Dilute BD matrigel and serum-free medium at a ratio of 1:8 and add to the upper chamber of the transwell for incubation. Serum-free medium was added to the upper chamber. Pre-chilled ethanol was fixed and 0.1 % crystal violet was used for staining. Finally, the chambers were removed, properly air-dried, and photographed for counting under the microscope.

### 2.9 Tumor xenograft assay

The experimental animals used in this experiment were 4-week-old BALB/c nude mice, purchased from Chengdu Dashuo Experimental Animal Co. The experimental production license number was SCXK(Chuan)2020-030. CRC cell line SW1116 was grown to logarithmic phase, digested, resuspended, and all adjusted and diluted to a concentration of 3 × 10^6^/mL. CRC cells of the sh-NC group and the sh-GDI2 group were grown subcutaneously on both sides of nude mice, respectively. SW1116 cells of the sh-NC group were injected on the left side and SW1116 cells of the sh-GDI2 group were injected on the right side. Six transplanted tumors were formed on each side, for a total of 12 tumors. The long and short diameters of the tumors were measured with vernier calipers and recorded on days 7, 14, 21, and 28 from the beginning of inoculation, respectively. Tumor volume = π/6 × a × b^2^ (a = length, b = wide). After 28 days, the nude mice were removed from the neck and executed, and the subcutaneous tumors were separated and weighed. Tumor tissues were stored in a −80 °C refrigerator and used for subsequent studies. Animal experiments were conducted in strict compliance with the Guide for the Management and Use of Laboratory Animals and were approved by the Experimental Animal Ethics Committee of Guangdong Medical University (No. GDY2202366).

### 2.10 Immunohistochemistry

The collected tissues were fixed in formalin for 24 h. The collected CRC tumor tissues were then paraffin-embedded and sectioned. Before the start of staining, the sections were subjected to xylene dewaxing, gradient alcohol hydration, citric acid high-pressure repair, hydrogen peroxide treatment, and BSA antigen closure in sequence. The corresponding primary antibody (ki-67, GB121141, Servicebio, 1:100; GDI2, GB121141, protein tech, 1:200) was added and incubated overnight, followed by secondary antibody (GB23301, GB23303, Servicebio, 1:5000) and incubated for 30 min. At the end of the reaction, the sections were washed 3 times with PBS. Finally, freshly prepared DAB was used for color development and hematoxylin re-staining. The sections were dehydrated and transparent in gradient alcohol and xylene in turn and then sealed. The sections were observed under a microscope (BA410, McArdy Industries Ltd.) and images were captured using a microscopic camera system. The percentage of positive area per image (%DAB Positive Tissue) was calculated using the Halo Data Analysis System.

### 2.11 Transcriptomic analysis

Total RNA was extracted from CRC cells in the sh-NC and sh-GDI2 groups, respectively. The mRNA was enriched in Oligo dT, then fragmented and synthesized into cDNA, ligated into an adaptor, and finally sequenced on the Illumina platform. The transcriptome detection and analysis were performed with the assistance of Beijing NovoTech Technology Co. The raw data of transcriptome sequencing were filtered to obtain high-quality data information. The genes were defined as differentially expressed genes with |log_2_FC|≥2 and FDR<0.05. The mRNAs in the samples were screened for differential genes. The IDs of differential genes were queried through the Bioconductor database, and the relevant installation packages of the Bioconductor platform were installed using the R language. Set P = 0.05, Q = 0.05, and perform GO enrichment analysis and KEGG enrichment analysis. Output the results and barplot histograms were plotted and bubble plots.

### 2.12 Protein interaction analysis

The possible target proteins bound by the GDI2 protein were predicted by Hitpredict online software. Based on the prediction results, the correlation between target proteins and the p53 pathway and TP53 protein was further analyzed by the TCGA database and String database. The binding of the target proteins to the GDI2 protein was verified in combination with Co-Immunoprecipitation (Co-IP) assay.

Total protein samples from CRC cells were extracted and lysed, and the total protein concentration of the lysates was measured using the BCA protein quantification kit (P0009, Beyotime, China). To Spin columns with End caps at the lower end, total protein was added, along with specific antibodies (GDI2, JC803862, abmart; RAB5A, #46449, CST) and Incubation buffer. The same amount of Control IgG was added to another group as a negative control. Incubate overnight at 4 °C. Add Protein A sepharose beads slurry to Spin columns to precipitate the immune complexes and incubate for 4 h with rotation. Remove the End caps and discard the supernatant. Wash the precipitated complexes with 1 × Washing buffer. After washing, Spin columns are centrifuged in Collection tubes and the Collection tubes and centrifugation products are discarded. Spin columns are placed in new EP tubes to collect the eluted product. The precipitated complexes are eluted with Elution buffer and the product is collected by centrifugation. Finally, Alkali neutralization buffer and 5 × Sample Buffer were added to the eluted product and heated in a boiling water bath. The final IP samples were separated by SDS-PAGE and the proteins were transferred to PVDF membranes for Western blot analysis using detection antibody hybridization and HRP-conjugated protein A secondary antibody at dilutions of 1:1500–1:3000.

### 2.13 Flow cytometry

Flow cytometric analysis was performed to determine the cell cycle phase distribution. HCT116 and SW1116 cells were inoculated on plates and trypsin digestion was performed after PBS washing. The appropriate amount of medium was added to become a single-cell suspension and counted. After the cells were attached to the wall, transfection was performed according to different groups. After 48 h of incubation, the supernatant was aspirated and discarded to obtain cell precipitates, which were fixed by adding 75 % pre-cooled ethanol and washed again with PBS. The cells were stained with PI, and then 10 μg/ml of RNase A was added for 30 min at room temperature and protected from light. Finally, the data collected from the flow cytometer (cytoflex, Beckman, American) were processed by ModiFit LT5.1 cell cycle analysis software.

### 2.14 Western blot

CRC cells were inoculated on the plate. Add RIPA cell lysis buffer to lyse cells and centrifuge to collect protein supernatant. Add SDS loading buffer and boil it in boiling water. Used SDS-PAGE gel electrophoresis to separate protein, transfer it to PVDF membrane, and then the membrane was blocked with 5 % non-fat dry milk in TBST at room temperature. After the incubation of primary antibody (GDI2, proteintech, 1:5000; p21, Abclonal, 1:2000; p53, Abclonal, 1:2000; p-p21 Affinity, 1:2000; p-p53, Abclonal, 1:2000; RAB5A, CST, 1:1000; β-actin, Abclonal, 1:50000), PBS was washed three times and secondary antibody (AS014, abclonal, 1:5000) was added for incubation. After PBS washed, the luminous solution was added and an ECL chemiluminescence system was used to observe the final results.

### 2.15 Statistical analysis

The data were analyzed with GraphPad Prism 8 (GraphPad Software, USA) and are presented as the means ± SD. Comparisons between the two groups were performed using Student's t-tests or one-way ANOVA. Differences with P-values of less than 0.05 were considered statistically significant.

## 3 Results

### 3.1 Differential expression and impact of GDI2 in CRC

**Fig. 1:**
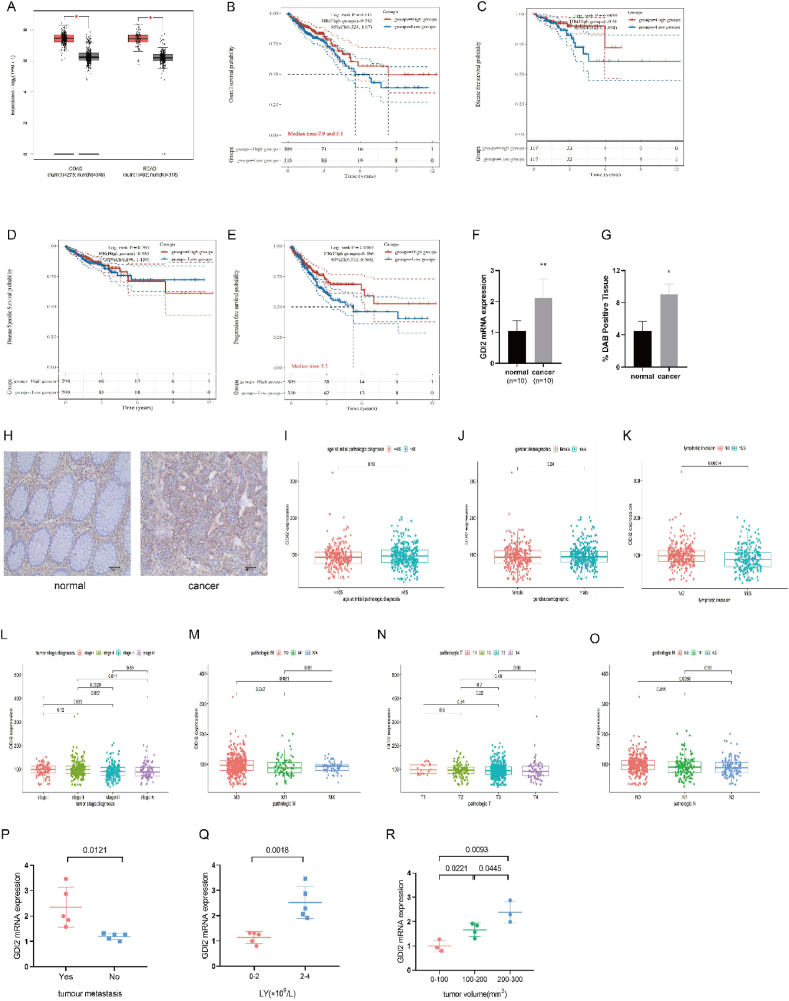
Analysis of GDI2 expression in CRC. A: TCGA database analysis of GDI2 mRNA expression differences in tumor tissues and paracancerous tissues of CRC patients. B-E: KM survival curves of GDI2 mRNA and OS, DFS, DSS, and PFS in TCGA data. Different groups were tested by log-rank. HR (High groups) represents the risk coefficient of the high expression group relative to low expression group samples, if HR > 1 means the mRNA is a risk factor, if HR < 1 means the mRNA is a protective factor. 95 % CL represents the HR confidence interval. F: qRT-PCR to detect GDI2 mRNA expression levels in normal tissues adjacent to cancer and CRC tissues. G-H: Immunohistochemical detection of GDI2 expression levels in normal tissues adjacent to cancer and CRC tissues ( × 20). ∗*P* < 0.05, ∗∗*P* < 0.01 compared with normal tissues next to cancer. I-O: TCGA database to analyze the correlation between GDI2 expression levels and age, gender, lymphatic count, tumor stage, M stage, T stage, and N stage of CRC patients. P-R: Clinical information to analyze the correlation between GDI2 expression level and the presence of distant metastasis, lymphocyte count, and tumor volume size in CRC patients.

GDI2 mRNA expression in CRC tissues was analyzed using the TCGA database. The expression of GDI2 was greater in tumor tissues of colon cancer patients or rectal cancer patients than in normal tissues (*P* < 0.05) (Fig. 1A), and it is hypothesized that GDI2 plays an important role in the proliferation and development of human CRC. The clinical prognostic relationship between GDI2 mRNA expression levels and overall survival (OS), progression-free survival (PFS), disease-free survival (DFS), and disease-specific survival (DSS) of CRC patients was examined by the GEPIA data analysis website to assess the relationship between GDI2 expression and survival of CRC patients in the TCGA database. The results of Kaplan-Meier survival curves showed (Fig. 1B–E) that the disease-specific survival of patients with low GDI2 expression was better than that of patients with high expression. There was a significant difference between the progression-free survival of the two groups of patients (*P* < 0.05).

GDI2 expression levels in clinical samples were detected by qRT-PCR and immunohistochemical assays. The qRT-PCR results showed (Fig. 1F) that the mRNA expression level of GDI2 in CRC tumor tissues was higher than that in normal tissues adjacent to the cancer (*P* < 0.01). In the results of immunohistochemistry experiments (Fig. 1G and H), the expression of GDI2 in tumor tissues was significantly higher than that in normal tissues adjacent to cancer (*P* < 0.05). The correlation between GDI2 expression levels and clinicopathological indices of CRC patients was analyzed by the TCGA database and clinical information. Among them, the results of TCGA data analysis showed (Fig. 1I–O) that the high expression of GDI2 was not significantly correlated with age (*P* = 0.13), gender (*P* = 0.24), lymphatic count (*P* > 0.05) and T-stage (*P* > 0.05). However, there were significant correlations with lymphatic metastasis (*P* = 0.00014), M0/M1 stage (*P* = 0.047), N0/N1 (*P* = 0.011) stage, and tumor stage (*P* < 0.05). The results of clinical information analysis showed (Fig. 1P–R) that the expression level of GDI2 was significantly correlated with the presence of metastasis (*P* = 0.0121), lymphocyte count (*P* = 0.0018), and tumor volume size (*P* < 0.05).

### 3.2 Silencing GDI2 inhibits the proliferation, migration, and invasion of CRC cells

**Fig. 2:**
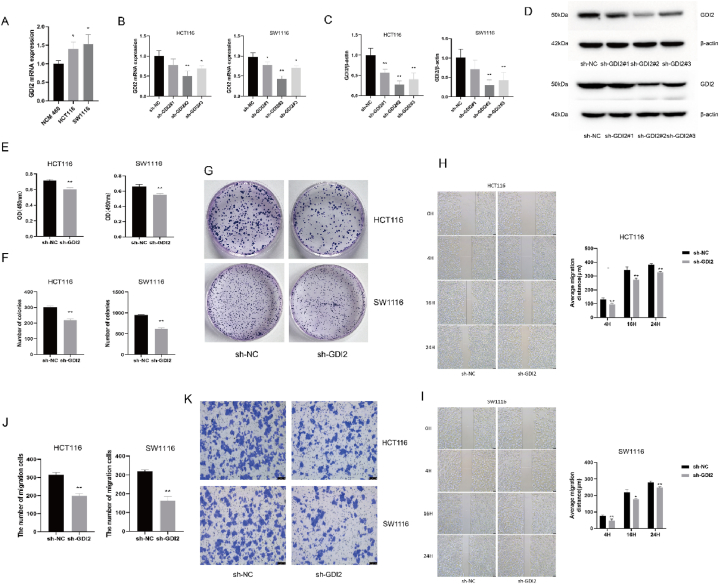
Effect of GDI2 silencing on proliferation, migration, and invasion of CRC. A: qRT-PCR to detect the difference of GDI2 mRNA expression in normal colonic epithelial cells and CRC cells. B: qRT-PCR to detect GDI2 mRNA expression levels in CRC cells after treatment with different shRNA sequences. C-D: Western blot detection of GDI2 protein expression levels in CRC cells after treatment with different shRNA sequences. E: CCK-8 assay to detect the effect of silencing GDI2 on the proliferation of CRC cells. F-G: Plate clone formation assay to detect the ability of CRC cells to form clones. H-I: The wound healing assay was used to detect the migration ability of CRC cells at 0 h, 4 h, 16 h, and 24 h ( × 50). J-K: Transwell invasion assay to detect the invasive ability of CRC cells ( × 40). Compared with the sh-NC group, ∗*P* < 0.05, ∗∗*P* < 0.01.

The difference in mRNA expression of GDI2 in CRC cells and normal colorectal epithelial cells was determined by qRT-PCR assay. The results showed that the mRNA expression levels of GDI2 in CRC cells (HCT116 and SW1116) were both higher than those in normal colonic epithelial cells NCM460 (*P* < 0.05) (Fig. 2A). In order to successfully investigate the effect of GDI2 on the development of CRC cells subsequently, HCT116 cells and SW1116 cells were used to screen the most suitable shRNA for subsequent study by silencing the expression of GDI2 mRNA in the cells. In CRC cells, the GDI2 mRNA expression level and protein expression level of cells in the sh-GDI2#2 group were lower than those in sh-GDI2#1 and sh-GDI2#3 groups, and significantly lower than those in the sh-NC group (*P* < 0.01) (Fig. 2B–D). Therefore, GDI2#2 was selected as the most suitable shRNA for the follow-up experiments.

In the CCK-8 assay, the OD450 values of cells in the sh-GDI2 group were all significantly lower than those of CRC cells in the sh-NC group (*P* < 0.01) (Fig. 2E). Silencing of GDI2 mRNA was able to reduce the proliferative activity of CRC cells HCT116 and SW1116 cells. The results of the plate clone formations assay are shown in Fig. 2F and G. The number of clone formation in the sh-GDI2 group was smaller than that in the sh-NC group for both HCT116 cells or SW1116 cells (*P* < 0.01). The results of wound healing experiments are shown in Fig. 2H and I. Compared with the cells in the sh-NC group, the mean migration distance of both HCT116 cells and SW1116 cells in the sh-GDI2 group was significantly reduced (*P* < 0.05, *P* < 0.01) in 4 h,16 h, and 24 h, and the migration ability was decreased. The number of invasions of HCT116 and SW1116 cells was also significantly reduced (*P* < 0.01) compared to the sh-NC group after silencing GDI2 mRNA (Fig. 2J and K), and the invasion ability of CRC cells was diminished. The above results demonstrated that silencing GDI2 inhibited the proliferative activity, migration, and invasive ability of CRC cells.

### 3.3 Silencing GDI2 inhibits CRC xenograft tumor proliferation

**Fig. 3:**
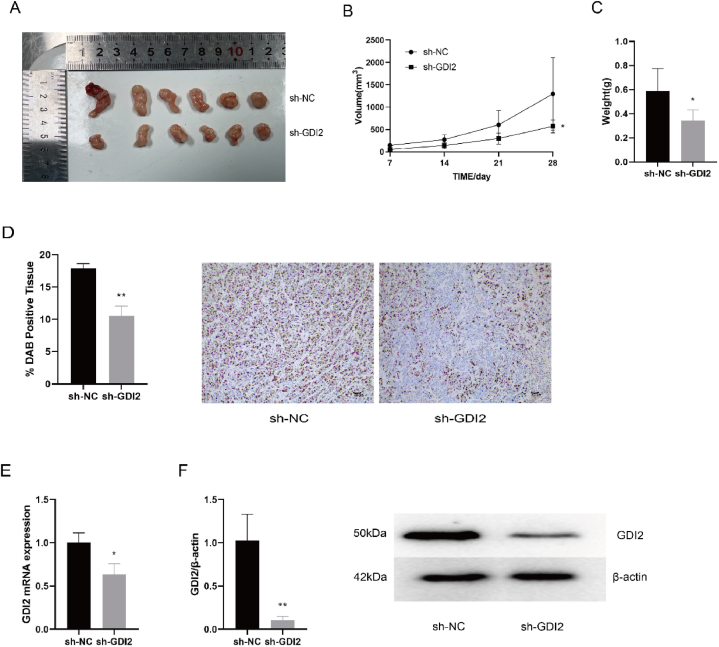
Effect of silencing GDI2 on CRC xenograft tumors. A: Tumor plot of dissected xenograft tumors. B: Growth curves of CRC transplant tumors during the trial. C: Weight of xenograft tumors. D: Immunohistochemical detection of ki-67 expression level of tumors ( × 10). E: qRT-PCR detection of GDI2 mRNA expression level of tumors. F: Western blot detection of GDI2 protein expression of tumors. Compared with the sh-NC group, ∗*P* < 0.05, ∗∗*P* < 0.01.

The tumor diameter of transplanted tumors in nude mice was measured on days 7, 14, 21, and 28 after inoculation with CRC cells SW1116. At the end of the experiment, the mice were sacrificed using the cervical dislocation method and executed and all subcutaneous transplanted tumors were photographed as in Fig. 3A. The volume and mass of the transplanted tumors in the sh-GDI2 group were significantly smaller than those in the sh-NC group. The tumor volume was calculated based on the tumor diameter, and the growth curve of the transplanted tumors was plotted in Fig. 3B. It can be seen that the volume growth trend of transplanted tumor in the sh-GDI2 group of nude mice was lower compared with that of the transplanted tumor in the sh-NC group (*P* < 0.05). The results of the statistical analysis of the final transplanted tumor weight are shown in Fig. 3C. The tumor weight of the transplanted tumor in the sh-NC group was (0.589 ± 0.168) g, and the tumor weight of the transplant tumor in the sh-GDI2 group was (0.342 ± 0.083) g, which was lower than the sh-NC group (*P* < 0.05). It is suggested that silencing the GDI2 mRNA has an inhibitory effect on the growth of CRC xenograft tumors.

The expression levels of ki-67 in xenograft tumors were detected by immunohistochemical staining (Fig. 3D). The expression level of ki-67 in the tumors of the sh-GDI2 group was remarkably lower than that of the sh-NC group (*P* < 0.01), which means that the proliferation ability of the tumors was reduced. The results of qRT-PCR experiments and Western blot experiments are shown in Fig. 3E and F. The mRNA expression level of GDI2 in transplanted tumors of sh-GDI2 group was lower than that of transplanted tumors of the sh-NC group (*P* < 0.05), and the protein expression level was significantly lower than that of the sh-NC group (*P* < 0.01). In conclusion, silencing the GDI2 mRNA inhibited the proliferation development of CRC tumors in vivo.

### 3.4 Silencing of GDI2 induces cell cycle arrest in CRC

**Fig. 4:**
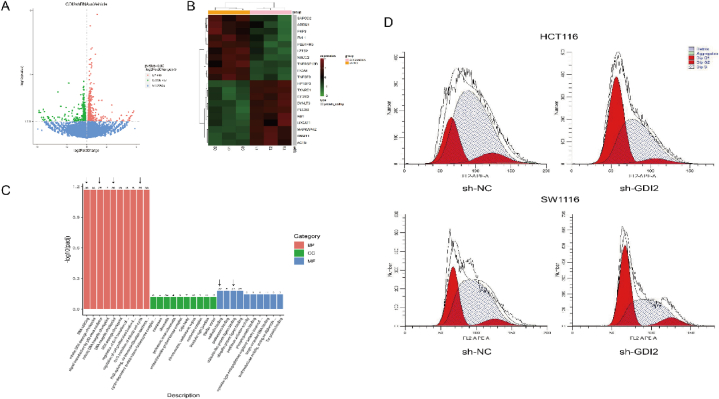
Exploration of the mechanism by which silencing of GDI2 affects CRC. A: Volcano plot of differentially expressed genes after silencing GDI2 gene in CRC cells (red shows up-regulated genes, green shows down-regulated genes). B: Heat map of Top10 down-regulated differential genes and Top10 up-regulated differential genes. C: GO functional enrichment analysis of differentially expressed genes histogram. D: Flow cytometry detection of CRC cell cycle arrest after silencing GDI2.

The possible mechanism of GDI2 gene action in CRC cells was analyzed based on transcriptomics. The distribution of differentially expressed genes was reflected by the volcano plot (Fig. 4A). The total number of differentially expressed genes obtained was 855, with 448 up-regulated genes and 407 down-regulated genes. Fig. 4B shows the top ten down-regulated differentially expressed genes and the top ten up-regulated differentially expressed genes. C1-C3 are the control CRC cells, T1-T3 are the sh-GDI2 group CRC cells, and the vertical coordinates indicate the normalized values of differential genes FPKM. Among the down-regulated differential genes, ADRM1 and ITGA5 were associated with CRC. Among the up-regulated differential genes, TXNRD1, EIF2S2, PEG10, and RB1 were associated with CRC.

To investigate the biological processes involved in GDI2 in CRC cells, GO functional enrichment analysis was performed on differentially expressed genes. From the results of the GO enrichment analysis, the most significant 30 items were selected to draw a bar graph for display (Fig. 4C). Among them, the significantly differentially expressed genes were mainly involved in RNA splicing, signal transduction by p53 class mediator, cadherin, ubiquitin-like protein ligase binding, G1/S transition of the mitotic cell cycle, DNA integrity, and other biological processes. This suggests that GDI2 may be involved in regulating the development of CRC through the above biological processes. To verify the effect of GDI2 expression on the cell cycle, the cell cycle phase distribution of each group was detected by flow cytometry, and the analysis results were shown in Table 1, Table 2, and Fig. 4D. Compared with the sh-NC group, the percentage of G0/G1 phase cells in CRC cells in the sh-GDI2 group was increased (*P* < 0.01). Meanwhile, the percentage of S-phase cells in CRC was significantly decreased in the sh-GDI2 group (*P* < 0.01). It demonstrated that CRC cells were significantly stalled in the G1 phase after silencing GDI2, which was consistent with the results of GO functional enrichment analysis.

**Table 1:** Effect of different groups on the cell cycle of HCT116 cells($\bar{x}$ ±standard deviation; n = 3).

Group	G0/G1 phase	G2/M phase	S phase
sh-NC	18.66 ± 1.07	9.38 ± 0.62	71.96 ± 0.61
sh-GDI2	44.04 ± 1.52^a^	6.30 ± 2.19	49.66 ± 1.27^a^

Note: Compared with sh-NC group.

a *P* < 0.01.

**Table 2:** Effect of different groups on the cell cycle of SW1116 cells($\bar{x}$ ±standard deviation; n = 3).

Group	G0/G1 phase	G2/M phase	S phase
sh-NC	27.47 ± 0.89	6.73 ± 0.48	65.79 ± 0.97
sh-GDI2	44.04 ± 0.64^a^	8.76 ± 0.47^a^	47.18 ± 1.10^a^

Note: Compared with sh-NC group.

a *P* < 0.01.

### 3.5 Silencing GDI2 targets binding to RAB5A protein to inhibit CRC

**Fig. 5:**
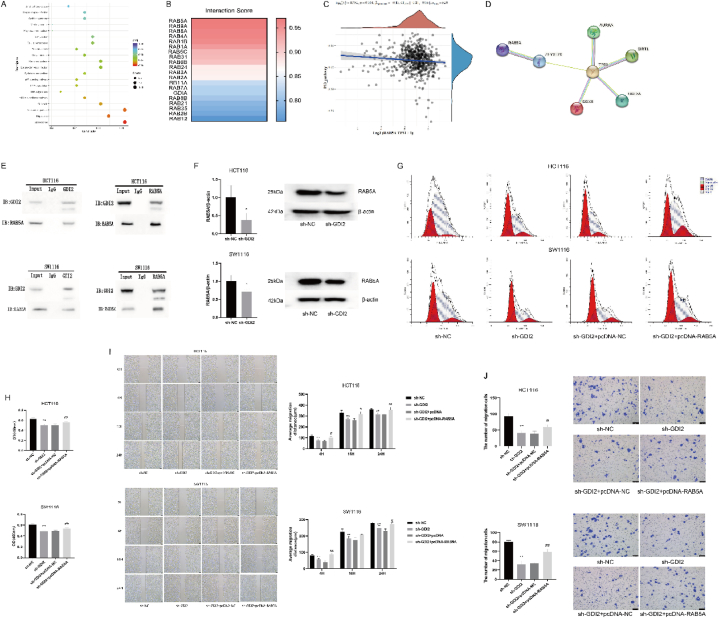
Effect of overexpression of RAB5A on CRC when GDI2 was silenced. A: Scatter plot of KEGG pathway enrichment analysis of upregulated differentially expressed genes. B: Results predicted by Hitpredict. C: Correlation between RAB5A protein and p53 pathway analyzed by TCGA database. D: Protein interaction network analysis of RAB5A and TP53 by String database. E: Immunoprecipitation assay to verify the presence of GDI2 protein binding to RAB5A. F: WB detection of the effect of silencing GDI2 on RAB5A protein expression. G: Flow cytometry to detect the effect of overexpression of RAB5A on cell cycle when GDI2 was silenced. H: CCK-8 assay to detect the effect of overexpression of RAB5A on cell cycle when GDI2 was silenced. I: Wound healing assay to detect the effect of overexpression of RAB5A on CRC cell migration when GDI2 was silenced ( × 50) (0 h, 4 h, 16 h, and 24 h). J: Transwell invasion assay to detect the effect of overexpression of RAB5A on CRC cell invasion when GDI2 was silenced ( × 40). Compared with the sh-NC group, ∗*P* < 0.05, ∗∗*P* < 0.01; compared with the sh-GDI2 group, ^#^*P* < 0.05, ^##^*P* < 0.01.

The results of KEGG pathway enrichment analysis showed (Fig. 5A) that upregulated differentially expressed genes were significantly enriched in tight junctions and cellular senescence pathways, and significantly enriched in the p53 signaling pathway. It is suggested that the p53 signaling pathway is one of the key pathways of GDI2 affecting the development of CRC cells. The Hitpredict software was used to predict the possible target proteins bound by the GDI2 protein and to investigate the relationship between target proteins and p53 to further validate the effect of GDI2 on the p53 signaling pathway. Hitpredict software predicted 130 target proteins, among which the 20 proteins with the highest interaction scores are shown in Fig. 5B. The RAB5A protein had the highest interaction score, so RAB5A was selected for the follow-up study. Spearman correlation analysis between RAB5A and p53 pathway score was performed according to the TCGA database. The results showed *P* = 0.0001, and there may be a high correlation between RAB5A and the p53 pathway (Fig. 5C). The interaction between TP53 and RAB5A was analyzed by the String database (Fig. 5D), and the results showed that TP53 could interact with RAB5A protein through ZFYVE20.

The binding of GDI2 to RAB5A was verified by Co-IP assay. The results showed that RAB5A protein was present in the enriched GDI2 protein and GDI2 protein was also present in the enriched RAB5A protein in both HCT116 cells and SW1116 cells (Fig. 5E). There was mutual binding between the GDI2 protein and RAB5A protein. The results of Western blot analysis demonstrated that the expression levels of the RAB5A protein in HCT116 cells and SW1116 cells were lower than those in the sh-NC group after silencing GDI2 (*P* < 0.05) (Fig. 5F).

Cells were divided into the sh-NC group, sh-GDI2 group, sh-GDI2+pcDNA-NC group, and sh-GDI2+pcDNA-RAB5A group to detect the effects of overexpression of RAB5A on cell cycle, cell proliferation, migration and invasion when GDI2 was silenced. The percentage of cells stalled in the G0/G1 phase was compared between groups (Table 3, Table 4, and Fig. 5G). The percentage of CRC cells stalled at the G0/G1 phase was significantly increased in the sh-GDI2 group compared with the sh-NC group (*P* < 0.01). Compared with the sh-GDI2 group, there was no significant change in the sh-GDI2+pcDNA-NC group (*P* > 0.05), and the percentage of cells in the G0/G1 phase was significantly lower in the sh-GDI2+pcDNA-RAB5A group (*P* < 0.01). Overexpression of RAB5A inhibited the cell cycle arrest induced by silencing GDI2.

**Table 3:** Effect of different groups on the cell cycle of HCT116 cells($\bar{x}$ ±standard deviation; n = 3).

Group	G0/G1 phase	G2/M phase	S phase
sh-NC	19.96 ± 1.31	5.71 ± 2.67	74.33 ± 1.65
sh-GDI2	41.56 ± 0.50∗∗	7.64 ± 1.29	50.80 ± 1.01∗∗
sh-GDI2+pcDNA-NC	41.54 ± 0.54	7.69 ± 0.42	50.77 ± 0.67
sh-GDI2+pcDNA-RAB5A	28.36 ± 0.43^##^	14.73 ± 1.10^##^	56.91 ± 1.10^##^

Note: Compared with sh-NC group, ∗*P* < 0.05, ∗∗*P* < 0.01; compared with sh-GDI2 group, ^#^*P* < 0.05, ^##^*P* < 0.01.

**Table 4:** Effect of different groups on the cell cycle of SW1116 cells($\bar{x}$ ±standard deviation; n = 3).

Group	G0/G1 phase	G2/M phase	S phase
sh-NC	29.44 ± 0.37	5.98 ± 0.99	64.58 ± 0.62
sh-GDI2	45.68 ± 0.65∗∗	8.50 ± 0.22	45.82 ± 0.80∗∗
sh-GDI2+pcDNA-NC	45.18 ± 0.83	7.22 ± 1.19	47.60 ± 1.57
sh-GDI2+pcDNA-RAB5A	38.05 ± 0.23^##^	7.10 ± 0.89	54.85 ± 0.68^##^

Note: Compared with sh-NC group, ∗*P* < 0.05, ∗∗*P* < 0.01; compared with sh-GDI2 group, ^#^*P* < 0.05, ^##^*P* < 0.01.

The results of the CCK-8 assay showed (Fig. 5H) that the OD values of CRC cells in the sh-GDI2 group were all lower compared with the sh-NC group (*P* < 0.01). Cell proliferation activity was significantly increased in the sh-GDI2+pcDNA-RAB5A group compared with the sh-GDI2 group (*P* < 0.01). In the results of the wound healing assay (Fig. 5I), the healing distance of CRC cells in the sh-GDI2+pcDNA-RAB5A group was significantly increased compared with the sh-GDI2 group (*P* < 0.05, *P* < 0.01) at 4 h and 24 h. The changes in cell invasion ability were detected by Transwell assay (Fig. 5J). The invasion ability of HCT166 cells and SW1116 cells in the sh-GDI2+pcDNA-RAB5A group was significantly higher than that in the sh-GDI2 group (*P* < 0.05, *P* < 0.01). Silencing of GDI2 inhibited the proliferation, migration and invasion of CRC cells and induced cell cycle arrest, while overexpression of RAB5A increased the proliferation, migration, and invasion ability of CRC cells and inhibited cell cycle arrest.

### 3.6 Silencing GDI2 inhibits CRC through activation of p53 signaling pathway

**Fig. 6:**
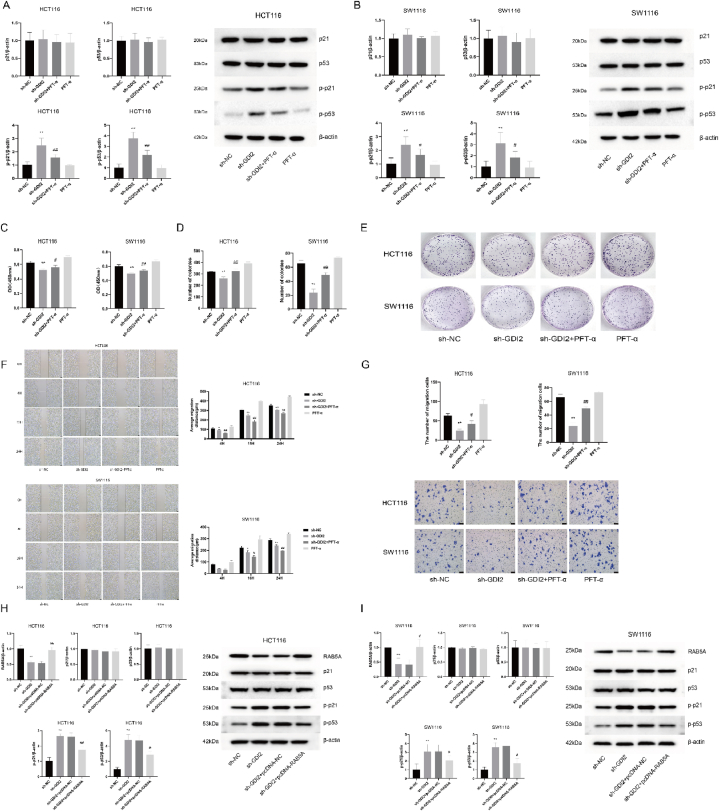
Silencing of GDI2 via p53 pathway affects CRC cells. A: WB detection of p21, p53, p-p21, and p-p53 protein expression in HCT116 cells. B: WB detection of p21, p53, p-p21, and p-p53 protein expression in SW1116 cells. C: CCK-8 assay to detect proliferative activity of CRC cells. D-E: Plate clone formation assay to detect CRC cell clone formation ability. F: The wound healing assay was used to detect the migration ability of CRC cells at 0 h, 4 h, 16 h, and 24 h ( × 50). G: Transwell assay to detect the invasive ability of CRC cells ( × 40). H: WB assay to detect the effect of overexpression of RAB5A on p53 pathway protein expression in HCT116 cells when GDI2 was silenced. I: WB detection of the effect of overexpression of RAB5A on p53 pathway protein expression in SW1116 cells upon silencing of GDI2. Compared with the sh-NC group, ∗*P* < 0.05, ∗∗*P* < 0.01; compared with the sh-GDI2 group, ^#^*P* < 0.05, ^##^*P* < 0.01.

CRC cells, HCT116 and SW1116, were divided into sh-NC, sh-GDI2, sh-GDI2+PFT-α, and PFT-α groups, respectively. The expression levels of p53 pathway-related proteins in CRC cells in the sh-GDI2 group were similar to those in the sh-NC group, and the expression levels of p-p21 and p-p53 proteins were significantly higher than those in the sh-NC group (*P* < 0.01). It indicates that silencing GDI2 mediates the activation of the p53 signaling pathway. Compared with the sh-GDI2 group, the p-p21 and p-p53 protein expressions in the sh-GDI2+PTF-α group were significantly downregulated (*P* < 0.05), and the p53 pathway inhibitor inhibited the activation of the p53 pathway by silencing GDI2 (Fig. 6A and B).

The results of CCK-8 experiments were shown in Fig. 6C. The OD450 values of CRC cells in the sh-GDI2 group were significantly decreased compared with the sh-NC group (*P* < 0.01). The OD450 values of HCT116 cells and SW1116 cells in the sh-GDI2+PFT-α group were increased compared with cells in the sh-GDI2 group (*P* < 0.05, *P* < 0.01). The results of the plate formation clone assay were shown in Fig. 6D and E, in which the number of clone formation in CRC cells in the sh-GDI2 group was significantly less than that in the sh-NC group (*P* < 0.01). Both HCT116 cells and SW1116 cells in the sh-GDI2+PFT-α group formed significantly more clones compared with cells in the sh-GDI2 group (*P* < 0.01). The results showed that at 16 h and 24 h, the mean migration distance of CRC cells in the sh-GDI2+PFT-α group was significantly greater than that of CRC cells in the sh-NC group (*P* < 0.05, *P* < 0.01) (Fig. 6F). In addition, the results of the Transwell invasion assay showed (Fig. 6G) that the number of CRC cells crossing Transwell chambers was significantly increased in the sh-GDI2+PFT-α group compared with cells in the sh-GDI2 group (*P* < 0.05, *P* < 0.01). After silencing the GDI2 gene, the proliferation, migration, and invasion ability of CRC cells were reduced, but the addition of the p53 pathway inhibitor restored the proliferation, migration, and invasion ability of CRC cells.

Western blot detected the effect of overexpression of RAB5A on the expression level of p53 pathway-related proteins upon silencing of GDI2 (Fig. 6H and I). Compared with the sh-GDI2 group, RAB5A protein expression levels were significantly higher in the sh-GDI2+pcDNA-RAB5A group (*P* < 0.01, *P* < 0.05). The expression levels of p-p21 and p-p53 proteins were significantly increased in the sh-GDI2 group compared with the sh-NC group (*P* < 0.01). The expression levels of p-p21 and p-p53 proteins were decreased in cells of the sh-GDI2+pcDNA-RAB5A group compared with the sh-GDI2 group (*P* < 0.05). Silencing of GDI2 mediated the activation of the p53 pathway, while overexpression of RAB5A conversely decreased p53 pathway activity, and overexpression of RAB5A exerted the same effect as the p53 pathway inhibitor.

## 4 Discussion

CRC is one of the most common malignancies. The prognostic outcome of CRC often correlates with the stage at which it is diagnosed, with survival rates higher for early diagnosis than for advanced stages, and much lower for patients who develop distant metastatic stages [[16], [17], [18]]. Although great progress has been made in recent years in the screening and treatment of CRC, the incidence, prevalence, and mortality of CRC remain high [19]. Therefore, the search for new CRC biomarkers and therapeutic targets is crucial to improve the prognosis and enhance the clinical management of CRC.

GDI2 belongs to a small family of chaperone proteins that are mainly expressed in hematopoietic, endothelial, and epithelial cells [20,21]. Abnormal expression of the GDI2 gene has been demonstrated in many cancer types. In recent studies in prostate cancer, GDI2 expression was found to be upregulated in prostate cancer cells and tissues. Knockdown of GDI2 inhibits cell proliferation but promotes apoptosis [8]. In thyroid cancer cells, microRNA-15b-5p can inhibit cell proliferation and invasion by targeting GDI2, which is associated with poor prognosis in thyroid cancer patients and is negatively regulated by miR-15b-5p [22]. The differential expression of GDI2 in gastric and hepatocellular carcinoma tissues provides potential insights for identifying biomarkers, predicting diagnosis, and assessing prognosis in gastric and hepatocellular carcinoma patients [9,23]. However, the expression profile and functional role of GDI2 in CRC have not been investigated. CRC possesses the common biological characteristics of most malignant tumors, including malignant proliferation, invasion, migration, and high levels of angiogenesis [24].In this study, we first examined clinical specimens by qRT-PCR assay and immunohistochemical assay, and based on bioinformatics analysis of the TCGA database, we investigated the expression differences and effects of GDI2 in CRC tissues. Our study demonstrated that silencing GDI2 in CRC cells reduces their proliferative activity, migration, and invasive capabilities as well as in vivo tumor growth inhibition. In addition, the possible mechanism of the effect of silencing GDI2 on CRC was further explored by transcriptomics.

Transcriptome analysis showed that silencing of GDI2 resulted in a total of 855 differential genes, of which 448 differential genes were upregulated and 407 differential genes. GO functional enrichment analysis showed that differentially expressed genes were significantly enriched in the biological process of cell cycle arrest. The cell cycle is usually divided into G1, S, G2, and M phases. Cell cycle blocks can be divided into three categories, including G1 phase DNA damage checkpoints, S phase DNA damage checkpoints, and G2 phase DNA damage checkpoints [25]. When DNA damage occurs in cells in the G1 phase, the G1 phase checkpoint prevents the initiation of the DNA replication phase so that the cells do not enter the S phase, thus preventing the damaged DNA from replicating [26]. In a study by Yong Jungkang [25], MHY2245 can trigger apoptosis by inducing cell cycle arrest and exhibits anti-CRC effects associated with DNA damage response. A study by Muhammad Akhtar Ali demonstrated that the deletion of the transcription factor regulator ZBEED6 reduced the growth rate of CRC cells HCT116 by affecting the cell cycle [27]. Transcriptome analysis revealed that GDI2 silencing leads to cell cycle arrest, particularly at the G1 phase, which is consistent with the observed inhibition of CRC cell proliferation and is associated with DNA damage response.

In the results of the KEGG pathway enrichment analysis, up-regulated differentially expressed genes were significantly enriched in the p53 signaling pathway, and silencing GDI2 may mediate the activation of the p53 signaling pathway in CRC cells. To further explore the mechanism of action of GDI2 activation of the p53 pathway, the target protein RAB5A, which binds to GDI2, was predicted and analyzed by Hitpredict online software, and the relationship between RAB5A and p53 was investigated. RAB5A belongs to the Ras family of G proteins, which mainly regulates vesicle transport [28] and is involved in the development of many human cancers, which can promote tumor proliferation and distant metastasis, including breast, liver, lung, pancreatic, and ovarian cancers [29,30]. RAB5A and GDI2 protein existed to bind each other and there was a high correlation between RAB5A and p53 pathway activation. The higher the level of RAB5A expression, the lower the p53 pathway score. The p53 signaling pathway was found to be upregulated upon GDI2 silencing, with RAB5A, a G protein, predicted to mediate this effect. Western blot results showed that the silencing of GDI2 expression was also followed by a decrease in RAB5A expression level and an increase in p53 pathway activity. RAB5A's role in vesicle transport and cancer progression was highlighted, and its expression inversely correlated with p53 pathway activity.

Further analysis highlighted the p53 signaling pathway as a key mediator of GDI2's effects in CRC. p53 oncoprotein is activated in response to various stress signals and coordinates many downstream responses, such as DNA repair, cell cycle arrest, and cellular senescence [31].

The role of p53 in cell cycle arrest has been extensively studied. For example, activation of the ATM/ATR pathway by mouse embryonic fibroblasts exposed to DNA damage leads to activation of p53 and subsequent G1 arrest [32]. p53 induces G1 arrest mainly through transactivation of p21Waf1/Cip1, a cell cycle protein-dependent kinase inhibitor, and targeted disruption of the p21Waf1/Cip1 gene impairs the G1/S checkpoint in MEFs. Both p53 and p21 are important oncogenes in vivo and are involved in some cell cycle regulation through post-translational modifications such as phosphorylation and ubiquitination [33]. Lacroix's study showed that p53 and p21 in the p53 signaling pathway are biologically active after phosphorylation and affect tumor cell proliferation, invasion and cell cycle, and other biological processes [34]. Western blot experiment showed that silencing GDI2 was found to activate the p53 pathway, potentially through the regulation of RAB5A, a protein that interacts with GDI2. This activation leads to increased phosphorylation of p53 and p21, crucial for cell cycle regulation and tumor suppression. In conclusion, our findings suggest that GDI2 is a promising biomarker and therapeutic target for CRC. Its upregulation in tumor tissues and its role in modulating the p53 pathway offers new avenues for improving CRC diagnosis and treatment strategies. Further research is needed to fully understand the mechanisms by which GDI2 influences CRC progression and to validate its potential as a clinical tool.

## Funding statement

None.

## Ethics approval and informed consent

Written informed consent was obtained from all donors who provided samples. The trial was approved by the Ethics Committee of the Medical Center of the Affiliated Hospital of Guangdong Medical University (No. YJYS2022237). All animal experimental procedures were reviewed and approved by the Experimental Animal Ethics Committee of Guangdong Medical University (No. GDY2202366).

## Data availability statement

The datasets used and/or analyzed during the present study are available from the corresponding author upon reasonable request.

## CRediT authorship contribution statement

**Wen-Ting Ou:** Writing – review & editing, Software, Resources, Project administration, Funding acquisition, Formal analysis. **Rong-Jian Tan:** Writing – original draft, Software, Methodology, Investigation, Data curation. **Jia-Wei Zhai:** Writing – original draft, Project administration, Methodology, Data curation. **Li-Jun Sun:** Project administration, Methodology, Data curation. **Fei-Peng Xu:** Software, Project administration, Methodology, Funding acquisition, Formal analysis. **Xian-Jin Huang:** Methodology, Investigation, Data curation. **Zhen-Hao Quan:** Project administration, Methodology, Data curation. **Cai-Jin Zhou:** Writing – review & editing, Supervision, Software, Resources, Funding acquisition.

## Declaration of competing interest

The authors declare that they have no known competing financial interests or personal relationships that could have appeared to influence the work reported in this paper.
